# Making the Matrix for a Seamless Crown

**Published:** 1901-03-15

**Authors:** J. H. Prothero

**Affiliations:** Chicago, Ills.


### MAKING THE MATRIX FOR A SEAMLESS CROWN.

BY J. H. PROTHERO, D.D.S., CHICAGO, ILLS.

In the past two or three years considerable interest has been manifested in the construction of crowns by the seamless method, and it is claimed that more aesthetic results can be obtained by this system than in any other way. I am not an enthusiast, nor do I believe it is the *only method* for producing artistic crowns.

Well fitting, serviceable and aesthetic crowns have been made by the band and swaged cusp system that are doing good service, and look as well or better than the average seamless crown. I think, however, that those who have had experience with both methods will admit that each has a place where it can be used to better advantage than the other.

It occurs to me, that the seamless system is most valuable in those cases of porcelain bridge-work where metal crowns are used as abutments or piers. Platinum crowns, constructed after this method, can be run through the furnace any number of times, without injury, since there are no joints to open.

The prosthetist who attempts to make a seamless crown must be able to carve the tooth he desires to reproduce. If unable to do this, some other method, perhaps, will enable him to secure better results. Often those who can carve a properly shaped plaster model have felt that the seamless method was unreliable, because of the difficulty met with in securing an accurate counterdie in which to swage the crown.

Porosity of the sides and bottom of the matrix is a defect most liable to occur in casting the fusible metal. This is due to the presence of moisture, or gases, in the plaster model over which the matrix is formed and which can not escape on account of the rapid crystallization of the metal. If the fusible alloy could be kept in a molten condition a sufficient time to permit the moisture to make its escape through the metal the matrix would be perfect. This, however, is not feasible with the ordinary appliances in use.

The usual method of depriving the plaster of moisture is to heat it in the flame of a Bunsen burner, or alcohol lamp, but this is seldom successful, as there is no way of ascertaining when the model is in proper condition to receive the metal.

The following plan is offered to you for consideration, and after a trial of almost a year I feel justified in saying that if the details are correctly carried out a perfect matrix can be secured in more than 95 percent of cases. It is presumed that all of you are more or less familiar with the manner of constructing the plaster model of the tooth that is to be reproduced, so this part of the method will receive no consideration.

The model, having been constructed and carved to proper shape, is now ready to dry out. The metal is placed in the ladle and melted, the plaster crown grasped in a pair of long handled wire pliers that close automatically and press into it until entirely immersed. A ladle of small diameter but quite deep is used in order that the metal may entirely cover the crown.

When first introduced, considerable bubbling occurs, owing to the rapid escape of the steam. Vibration is distinctly felt in the plier handles and also in the ladle handle, so long as any moisture remains. As soon as this ceases it is a certain indication that the plaster is dry and free from all moisture or gas that might cause trouble in casting. Three to five minutes' time is usually sufficient to bring about this condition and insure certain results.

It is now removed from the ladle and placed in position on base on which it is to rest while the matrix is being cast. If set on mouldine, care should be taken that the cervical end is not pressed into the clay, as this would reduce the depth of the matrix and consequently shorten the crown.

A better method is to shape the mouldine with a curve corresponding to the gingival curve of the band from buccal to lingual and cover it over with a sheet of thin paper. A small hole can be drilled in the cervical end of the plaster crown, into which a peg is fitted, the outer end projecting an eighth of an inch or so. The crown is now pressed down on the mouldine, the peg passing through the paper and into the clay, and acting as an anchorage to hold the crown firmly to its base. If the model is not held down firmly, the metal, owing to its greater specific gravity, will displace it and it will float to the surface. The object in using the paper over the surface of the mouldine is to prevent the metal from coming in contact with it, as the glycerine, with which the clay is mixed, has more or less affinity for moisture and readily takes it up from the air, and if any is present on the surface against which the metal is poured the result will probably be a faulty matrix. By covering the mouldine and attaching the crown in the manner just described, error from this source is obviated.

The metal ring is now set over the pattern crown, and the strips of pasteboard inserted in the ring slots, the metal allowed to cool somewhat, or until it begins to thicken slightly, and is then poured upon the occlusal surface of the crown and allowed to trickle slowly over the sides until the matrix is of sufficient thickness.

The metal should be poured as cool as possible and yet be sufficiently plastic to copy accurately the surface over which it is cast. When set, it is knocked out of the ring, cooled, and split apart and the pattern removed. As before stated, a perfect matrix can be secured in almost every instance, if the details have been carefully attended to.

By a slight modification, this method is applicable to the construction of counterdies for ordinary swaged cusp work.

The gold band, upon which has been carved the plaster cusps, is covered with a coating of whiting and alcohol, to protect it from contamination by the baser metal. It is immersed in the manner before described, and held under the surface until all moisture is driven off, then set into the clay about to the line of junction of the band with cusp, the ring placed in position, and the matrix poured.

A word of caution is necessary at this point. If any of the whiting, covering the band, should become displaced, the baser metal immediately unites with the gold to such an extent that a new band will frequently be required. The whiting is very easily disturbed, the slightest touch being all that is required to dislodge it, so that too much care can not be exercised to avoid displacing it in handling the crown in the molten metal. (Dr. Taggart suggests wetting the whiting with gum arabic water to make it stick.)

I have been experimenting with a varnish that would be easy to apply and more difficult to remove than the whiting, but up to the present time have nothing of special value to offer. An idea occurred to me the other day that may overcome the difficulty. This is to cover the entire band, after the cusp is carved with plaster, so as to protect it perfectly from the metal.

This covering need not be more than two millimeters thick, and it will in no way interfere with the escape of the moisture contained inside the band. It may, perhaps, require a trifle longer time to prepare the model for casting, since it is somewhat more bulky, but that is the only objection to its use. (Dr. Nyman suggests rubbing powdered soapstone into the plaster model to fill the pores and get a smooth casting.)

When dry, the plaster covering can be removed, the case imbedded in mouldine in the usual way and the matrix cast.

Most excellent counterdies of uniform density throughout, in which the finest lines and the sharpest cusps can be copied, may be produced after this method, if good judgment and care be exercised in the various steps.—*February Dental Review.*

